# Development of the Integrated Computer Simulation Model of the Intracellular, Transmembrane, and Extracellular Domain of Platelet Integrin α _IIb_ β _3_ (Platelet Membrane Glycoprotein: GPIIb–IIIa)

**DOI:** 10.1055/a-2247-9438

**Published:** 2024-02-29

**Authors:** Masamitsu Nakayama, Shinichi Goto, Shinya Goto

**Affiliations:** 1 Department of Medicine (Cardiology), Tokai University School of Medicine, Isehara, Japan

**Keywords:** platelet, integrin α _IIb_ β _3_, molecular dynamic simulation, GPIIb/IIIa

## Abstract

**Background** The structure and functions of the extracellular domain of platelet integrin α _IIb_ β _3_ (platelet membrane glycoprotein: GPIIb–IIIa) change substantially upon platelet activation. However, the stability of the integrated model of extracellular/transmembrane/intracellular domains of integrin α _IIb_ β _3_ with the inactive state of the extracellular domain has not been clarified.

**Methods** The integrated model of integrin α _IIb_ β _3_ was developed by combining the extracellular domain adopted from the crystal structure and the transmembrane and intracellular domain obtained by Nuclear Magnetic Resonace (NMR). The transmembrane domain was settled into the phosphatidylcholine (2-oleoyl-1-palmitoyl-sn-glycerol-3-phosphocholine (POPC)) lipid bilayer model. The position coordinates and velocity vectors of all atoms and water molecules around them were calculated by molecular dynamic (MD) simulation with the use of Chemistry at Harvard Macromolecular Mechanics force field in every 2 × 10 ^−15^ seconds.

**Results** The root-mean-square deviations (RMSDs) of atoms constructing the integrated α _IIb_ β _3_ model apparently stabilized at approximately 23 Å after 200 ns of calculation. However, minor fluctuation persisted during the entire calculation period of 650 ns. The RMSDs of both α _IIb_ and β _3_ showed similar trends before 200 ns. The RMSD of β _3_ apparently stabilized approximately at 15 Å at 400 ns with persisting minor fluctuation afterward, while the structural fluctuation in α _IIb_ persisted throughout the 650 ns calculation period.

**Conclusion** In conclusion, the integrated model of the intracellular, transmembrane, and extracellular domain of integrin α _IIb_ β _3_ suggested persisting fluctuation even after convergence of MD calculation.

## Graphical abstract

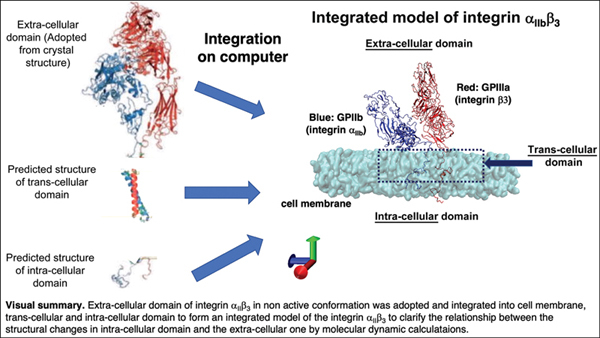

## Introduction

The integrin α _IIb_ β _3_ molecules known as platelet glycoprotein (GP) IIb/IIIa change their affinity to various plasma ligand proteins such as fibrinogen and von Willebrand factor (VWF) upon platelet activation. 1 Serious bleeding phenotype appears in patients deficient in the functions of α _IIb_ β _3_ , namely Glanzmann thrombasthenia 2 or fetal/neonatal alloimmune thrombocytopenia. 3 The functional blockage of integrin α _IIb_ β _3_ reduces the risk of thrombosis such as myocardial infarction but increases the risk of bleeding. 4 Thus, the function of integrin α _IIb_ β _3_ is essentially important for hemostasis and thrombus formation. A large body of studies have revealed that the mechanism of platelet activation depends on the functional changes in integrin α _IIb_ β _3_ . 1 The structural characteristics of both the extracellular domain that mediates biological function 5 6 7 and the intracellular domain that induce functional changes 8 9 10 11 were deeply investigated. Recently, Tong et al revealed the importance of an intermediate structure between active and nonactive conformation for platelet adhesion by the use of molecular dynamic (MD) calculations. 12 However, the stability of the structure of the integrin α _IIb_ β _3_ incorporated into the lipid membrane with the nonactive state of extracellular domain still needs to be elucidated.

Recent progress in computer technology and the evolutions of the force field incorporating quantum mechanics coarse-grained into molecular mechanics such as the CHARMM (Chemistry at Harvard Macromolecular Mechanics) enabled the construction of the biological functions of various proteins from the accumulations of simple physical movements of the atoms. 13 14 15 Various biological functions such as transmembrane water transportation were constructed by structural fluctuations of specific proteins such as aquaporin. 16 17 Specific biological functions of platelets such as adhesion on VWF under high shear stress conditions 18 19 were also simulated from dynamic movements of atoms. 20 21 The MD simulation calculation has also been applied in parts of integrin α _IIb_ β _3_ previously 22 and, recently, for whole molecules incorporated into lipid membrane by Tong et al. 12 Several previous studies revealed the important regions within the extracellular domain of α _IIb_ β _3_ to achieve its biological functions. 23 24 Moreover, the MD simulation was also applied to the intracellular domain of α _IIb_ β _3_ . 22 The integrated model of integrin α _IIb_ β _3_ constructed from intracellular, transmembrane, and extracellular domain was published recently. 12 Here, we have attempted to confirm the structural fluctuation of integrin α _IIb_ β _3_ incorporated into the lipid membrane.

We are proposing the hypothesis here that the structure of integrin α _IIb_ β _3_ is unstable as compared to other platelet glycoproteins such as GPIba even in the inactive conformation of extracellular domain.

## Material and Methods

### Molecular Dynamic Simulation

#### Initial Structure of GPIIb/IIIa

The initial structure of the extracellular domain of integrin α _IIb_ β _3_ was obtained from the previously published crystal structure representing the nonactivated conformation. 7 25 While the platelet membrane is known to contain phosphatidyl serine, 26 the cell membrane model composed from lipid bilayer 2-oleoyl-1-palmitoyl-sn-glycerol-3-phosphocholine (POPC) 27 was used in this study because the distributions of POPS were shown to be influenced substantially after platelet activation and the precise distributions of POPS before and after platelet activation have only been partly quantified. 28 The structure of the transmembrane and the intracellular domain was adopted from the previously published model predicted from NMR, electron cryo-microscopy, and single particle image reconstruction. 29 30 The POPC membrane model was settled at the transcellular domain of the integrated α _IIb_ β _3_ model. The integrated model of whole integrin α _IIb_ β _3_ was constructed according to the previously published conceptional model. 30

#### Molecular Dynamic Simulation Calculation

The water molecules were modeled as CHARMM transferable intermolecular potential with three interaction sites and were arranged around the atoms constructing the integrated model of α _IIb_ β _3_ according to the previous publication. 31 Newton's second law of F (force) = M (mass) × A (acceleration) was solved for all atoms constructing the integrated model of α _IIb_ β _3_ , lipid membrane, and water molecules. The calculation was conducted using NAnoscale Molecular Dynamics software 20 21 on a computer equipped with four NVIDIA Tesla V100 GPUs (HPC5000-XSLGPU4TS, HPC systems Inc., Tokyo, Japan). Since biological events occur in stable temperature and pressure, neither constant-temperature, constant-pressure ensemble (NPT) nor constant-temperature, constant-volume ensemble (NVP) ensembles were skipped. The position coordinates and velocity vectors of atoms and water molecules were calculated in each 2.0 femtosecond (10 ^−15^ s) using the CHARMM-36 force field. 32 33 The calculation started immediately from the initial structure. Visual molecular dynamics version 1.9.3 was used for the visualization of the results. 20 21

#### Root Mean Square Deviations

In each calculated structure, the average distances between various atoms excluding lipid bilayer and water molecules were calculated as the root mean square deviations (RMSDs) for all atoms constructing the integrated model of α _IIb_ β _3_ . To identify the specifically unstable regions within this calculation, the RMSDs were also calculated separately for α _IIb_ , in β _3_ , in the intracellular domain, in the transmembrane, and in the extracellular domains _._ The RMSDs were calculated every 10 picoseconds from the beginning to the end of the calculation.

The validity of calculation results was intuitively assessed by comparing the structure of extracellular domain of the integrated model of α _IIb_ β _3_ before and after MD calculation. The stability of RMSDs of atoms constructing the extracellular domain of the integrated model of α _IIb_ β _3_ within 20Å objectively confirms that the calculated structure is not extremely different from the crystal structure.

## Results

### Initial Structure

**Fig. 1:**
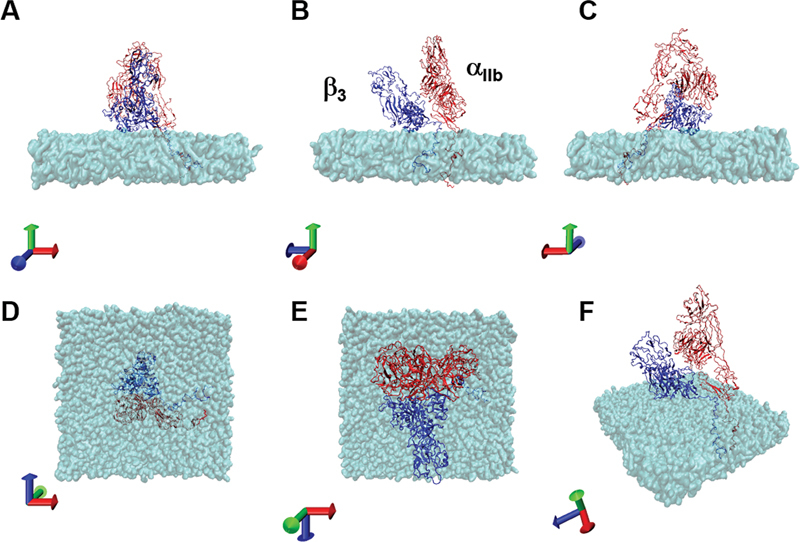
Initial structure of integrated model of integrin α _IIb_ β _3_ . The composed initial structure of integrin α _IIb_ β _3_ along with membrane bilayer are shown in each panel. The molecules constructing α _IIb_ and β _3_ are shown in red and blue, respectively. The membrane constructed from the bilayer of 2-oleoyl-1-pamlitoyl-sn-glyecro-3-phosphocholine is shown in light blue. The panel A to F show the view of the initial structure of the integrated model from the direction shown at left bottom of each panel.

Fig. 1 shows that the initial structure of the integrated model of integrin α _IIb_ β _3_ composed of the extracellular, transmembrane, and intracellular domain arranged within the POPC lipid bilayer. The protein structures are also provided as a pdb file as attached ( Supplemental pdb files 1 , available in the online version). The transmembrane domain is shown through a lipid bilayer.

### Structure after 700 ns of Molecular Dynamic Calculation



**Supplemental Movie A** Time-dependent changes in the structure of integrin α _IIb_ β _3_ from the frontal view.


**Supplemental Movie B** Time-dependent changes in the structure of integrin α _IIb_ β _3_ from the diagonal view.


**Fig. 2:**
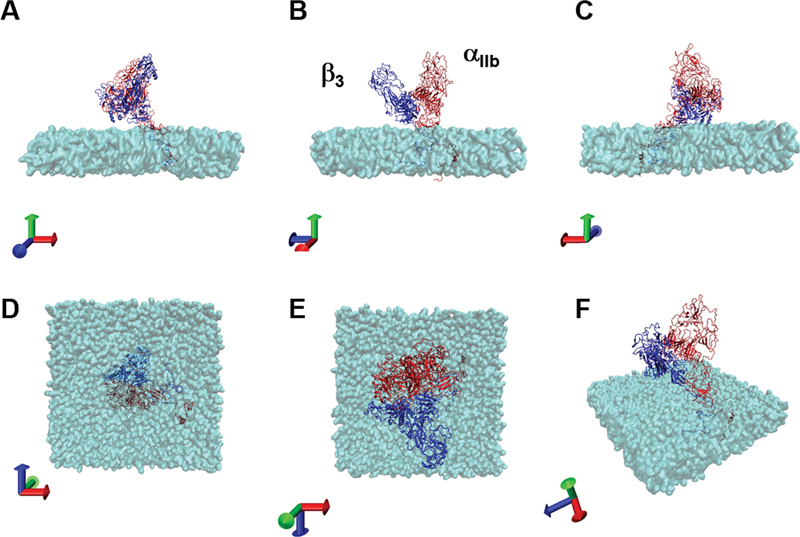
The structure of the integrated model of integrin α _IIb_ β _3_ . After 700 ns of calculation. The composed structure of integrin α _IIb_ β _3_ along with the membrane bilayer after 700 ns of MD calculation is shown. The panel A to F show the view of the initial structure of the integrated model from the direction shown at left bottom of each panel.

**Fig. 3:**
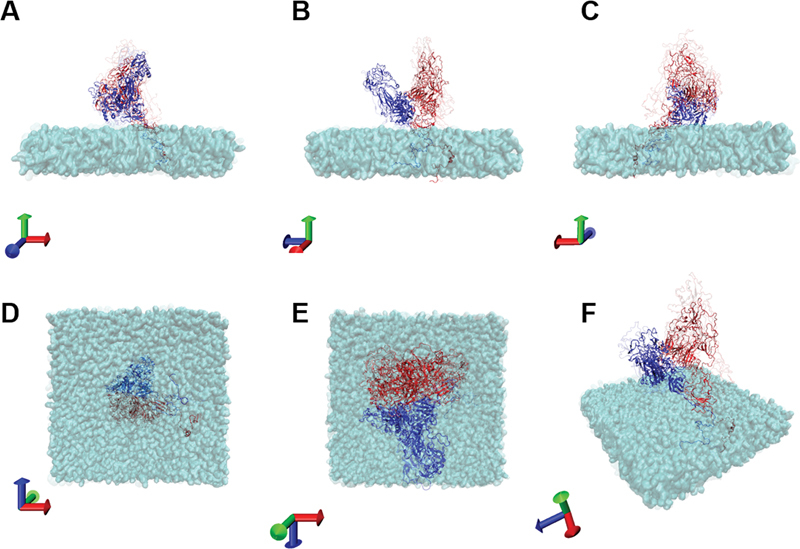
Overlayed images of initial structure and the one after 700 ns of calculations. The panel A to F are constructed by overlaying the images in the initial structure and the one after 700 ns of calculation.

Fig. 2 shows the structure of the integrated model of integrin α _IIb_ β _3_ after 700 ns (3.5 × 10 ^8^ step) of MD calculation. The protein structures provided as pdb files ( Supplemental pdb files 2 , available in the online version). There were apparent changes as compared to the initial structures. To further clarify the changes from the initial structure, each panels of Fig. 1 and Fig. 2 was overlayed to make Fig. 3 . The structural fluctuations of the integrated α _IIb_ β _3_ model from the beginning to the end of the calculation is summarized in two movies ( Supplemental movie A and Supplemental movie B , available in the online version). Apparently, the structural fluctuation was larger in α _IIb_ than in β _3._ As compared to the heavy chain, the light chain of α _IIb_ appeared most unstable.

**Fig. 4:**
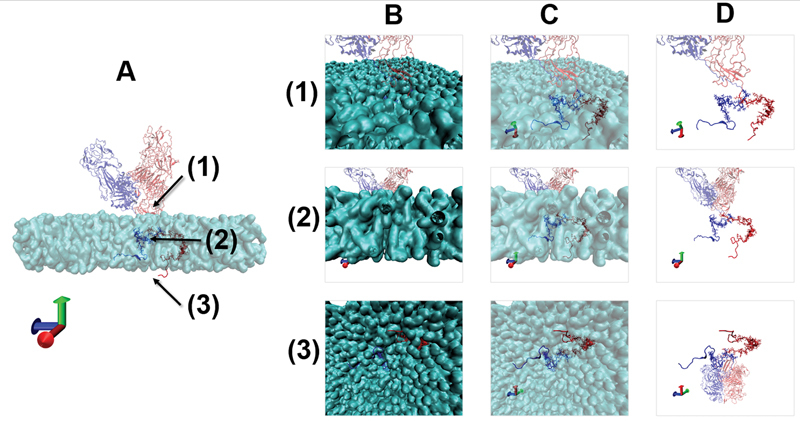
The detailed structure of the integrated model of integrin αIIbβ3 focusing transmembrane a domain after 700 ns of calculation. Panel A shows the overview of the calculation results at 700 ns. The arrow (1) indicates the base of the extracellular domain of integrin α _IIb_ β _3_ . The arrow (2) and (3) indicated the transmembrane and extracellular domain of integrin α _IIb_ β _3_ . Both the extracellular and intracellular domains are shown as ribbon diagram. The transmembrane domain is shown as ribbon diagram/ball and stick. Panel B, C, and D show the detailed structure of integrin α _IIb_ β _3_ around platelet membrane. (1), (2), and (3) correspond to the views shown in panel A. The lipid membrane was shown thick, transparent, and clear in panel B, C, and D. The red line indicated the structure of integrin α _IIb_ and the blue line indicates the structure of β _3_ .

Fig. 4 shows the detailed structure of the integrated model of integrin α _IIb_ β _3_ focusing on the transmembrane domain after 700 ns of calculation. The amino acid from 961 to 933 in α _IIb_ and 715 to 742 in β _3_ integrins are located within the lipid membrane.

### Root Mean Square Deviations

**Fig. 5:**
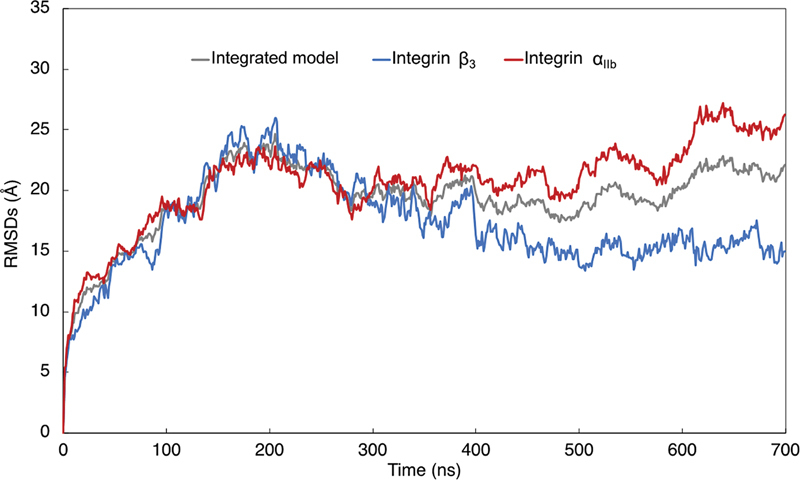
The root mean square deviations of atoms constructing the integrated model of integrin α _IIb_ β _3_ . The time-dependent changes in the root mean square deviations (RMSDs) of atoms constructing the whole integrated model of α _IIb_ β _3_ , excluding the water and the lipid, are shown as a gray line. The blue line represents the time-dependent changes in RMSDs in atoms constructing the β _3_ subunit in the integrin α _IIb_ β _3_ , while the red line represents that in α _IIb_ domain.

The calculated results of RMSDs in the integrated α _IIb_ β _3_ model and in the individual structure of α _IIb_ and β _3_ are shown in Fig. 5 . The RMSD of integrated α _IIb_ β _3_ model apparently stabilized at approximately 23 Å after 200 ns of calculation. However, minor fluctuation persisted until 650 ns to approximately 24 Å. The RMSD of both α _IIb_ (red line in Fig. 5 ) and β _3_ (blue line in Fig. 5 ) showed similar trends before 200 ns. The RMSD of β _3_ stabilized approximately at 15 Å at 400 ns, while it persisted to fluctuate until 650 ns in α _IIb_ .

**Fig. 6:**
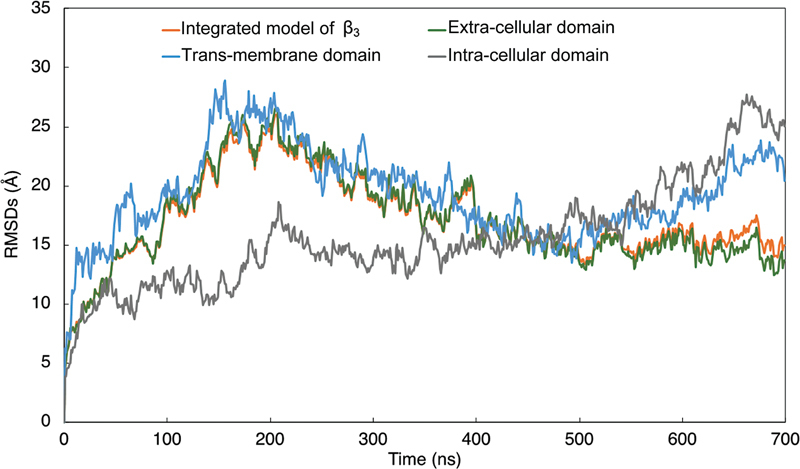
The root mean square deviations of atoms constructing β _3_ in the integrated model of integrin α _IIb_ β _3_ . The time-dependent changes in the root mean square deviations (RMSDs) of atoms constructing the β _3_ subunit in the integrated model of α _IIb_ β _3_ , excluding the water and the lipids, are shown as an orange line. The blue line represents the time-dependent changes in RMSDs in transmembrane domain of β _3_ molecule in the integrin α _IIb_ β _3_ . The time-dependent changes in RMSDs in the extracellular and the intracellular domains are shown in green and gray lines, respectively.

Fig. 6 shows the RMSDs in β _3_ within the integrated α _IIb_ β _3_ model. Overall, the RMSDs of β _3_ in the integrated α _IIb_ β _3_ model apparently stabilized at 15 Å after 600 ns of calculations. As compared to the extracellular domain, both intracellular and transmembrane domains were more unstable even after 500 ns of calculation.

**Fig. 7:**
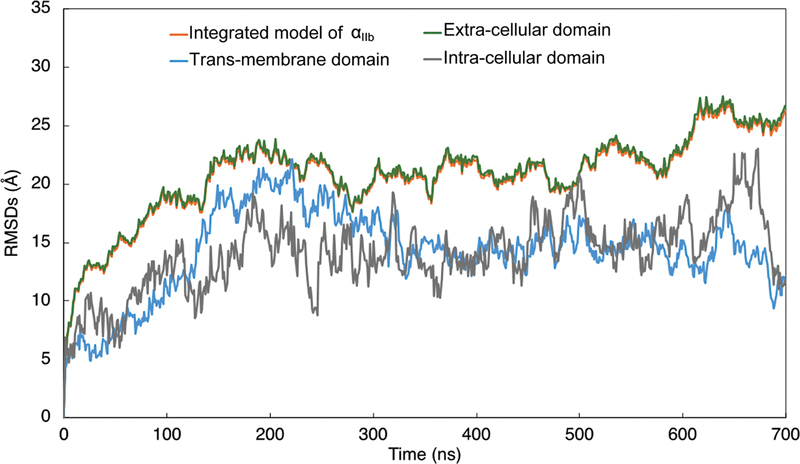
The root mean square deviations of atoms constructing α _IIb_ in the integrated model of integrin α _IIb_ β _3_ **.** The time-dependent changes in the root mean square deviations (RMSDs) of atoms constructing α _IIb_ in the integrated model of α _IIb_ β _3_ , excluding the water and the lipids, is shown as an orange line. The blue line represents the time-dependent changes in RMSDs in the transmembrane domain of β _3_ molecule in the integrin α _IIb_ β _3_ . The time-dependent changes in RMSDs in the extracellular and the intracellular domains are shown in green and gray lines, respectively.

Fig. 7 shows the RMSDs in α _IIb_ within the integrated α _IIb_ β _3_ model. The RMSDs of α _IIb_ in the integrated α _IIb_ β _3_ model did not stabilize even after 600 ns of calculation. The RMSD in α _II_ and extracellular domain were larger than that in their intracellular and transmembrane domains.

## Discussion



**The Supplemental Movie 1** Time-dependent change in the structure of the integrated model of integrin α _IIb_ β _3_ in frontal view excluding the water and lipid molecules. The results are expressed as the sequential snap-shot images obtained every 10 ns from the initial structure to the end of 700 ns.


**The Supplemental Movie 2** Time-dependent change in the structure of the integrated model of integrin α _IIb_ β _3_ in a diagonal view excluding the water and lipid molecules. The results are expressed as the sequential snap-shot images obtained every 10 ns from the initial structure to the end of 700 ns.


Integrin α _IIb_ β _3_ is one of the most commonly expressed platelet membrane GP. Unlike other commonly expressed pairs of protein complexes such as GPIb/IX, the biological functions of integrin α _IIb_ β _3_ change dramatically after platelet activation. The functional changes in integrin α _IIb_ β _3_ upon platelet activation are mediated mostly by the conformational changes in its extracellular domain. 34 Various ions including cations such as calcium and magnesium play important roles in keeping both inactive and active conformation of the extracellular domain of integrin α _IIb_ β _3_ . 35 36 37 The activated form of integrin α _IIb_ β _3_ can bind with ligand proteins such as fibrinogen and VWF although it could not bind them in its inactive form. The mechanisms of intracellular signaling pathways to achieve active conformation of integrin α _IIb_ β _3_ have deeply been investigated so far. Recently, the logical link between the structural changes in intracellular domain of α _IIb_ β _3_ on the substantial conformational changes in its extra-cellular domain was suggested by combining all-atom simulations, principal component analysis, and mesoscale modeling by Tong et al. 12 Here, the MD simulation of the integrated model of the extracellular, transmembrane, and intracellular domain of the integrin α _IIb_ β _3_ incorporated into the lipid bilayer membrane was conducted in all atoms constructing them. The structure of the integrated model continuously fluctuated even when the calculation was started from the inactive conformation of extracellular domain suggesting the structural instability of integrin α _IIb_ β _3_ even at the resting state.

As compared to other platelet membrane GPs such as GPIbα, the integrin α _IIb_ β _3_ model was structurally unstable even with a similar extent of calculation length. Indeed, the RSMD became apparently stable after the initial 200 ns of calculation but continued to fluctuate until 650 ns. The time-dependent fluctuation is clearer in the α _IIb_ domain than β _3_ . Within α _IIb_ , time-dependent fluctuation was clearer in the extracellular domain. For the future, we aim to apply this model to understand the logical link between the conformational changes in the intracellular domain in integrin α _IIb_ β _3_ induced by increased intracellular calcium ion concentration upon the activation of platelets on the conformational changes in its extracellular domain. 37

Molecular dynamic simulation is not a novel technic. 38 But, recent advances in high-performance computers enabled clarification of the specific biological functions by large-scale and long-time simulation calculation 39 such as water transportation by dynamic structural changes in specific proteins. 40 41 For the platelet membrane protein, the structural fluctuation and biological functions of platelet GPIbα binding with the A1 domain of VWF were extensively investigated. 20 21 42 Unlike the integrated α _IIb_ β _3_ model, RMSD of GPIbα binding with VWF converged to approximately 2 Å and stabilized after several hundred nanoseconds of calculation. As compared to GPIbα, the structure of integrin α _IIb_ β _3_ was apparently unstable as shown by the attached movies. Our MD calculation results are in agreement with the previous publication. 12 The substantial difference in the stability of the structure in commonly present platelet membrane GPIbα and GPIIb/IIIa of integrin α _IIb_ β _3_ is suggested.

Platelet activation initiated by various receptor stimulations rapidly increases the intracellular calcium ion concentration ([Ca ^2+^ ] _*i*_ ). The activation-dependent changes in the structure of the extracellular domain of the integrin α _IIb_ β _3_ occur subsequently to this. It is of note that active conformation of the extracellular domain of integrin α _IIb_ β _3_ rapidly reversed to the inactive state without continuous stimulation of the P2Y _12_ ADP receptor that is necessary for the cyclic increase in [Ca ^2+^ ] _*I*_ . 37 43 These experimental findings suggest that the changes in the structure of the extracellular domain of integrin α _IIb_ β _3_ are reversible events. Yet, the precise mechanism is still to be elucidated. Our computer simulation calculation findings that the structure of integrin α _IIb_ β _3_ in nature is not as stable as other membranous proteins such as GPIbα do not contradict with these previous findings. Various intracellular proteins such as talin 8 44 45 and kindlin 11 play a role in achieving and maintaining the active conformation of the extracellular domain of α _IIb_ β _3_ . The dynamic structural regulation process should be controlled by a cyclic increase in [Ca ^2+^ ] _*i*_ . Most likely, these intracellular proteins cause structural change in intracellular domain of integrin α _IIb_ β _3_ . Our integrated model of integrin α _IIb_ β _3_ started from the inactive conformation of extra-cellular domain. We have shown here the structural fluctuation of our model even within the inactive conformation of extracellular domain. These structural fluctuations may explain the redundant biological function of integrin α _IIb_ β _3_ including its binding capacity to bind to fibrinogen with RGD (arginine–glycine–aspartate) and NGD (asparagine–glycine–arginine) peptides. 46 In the future, we are aiming to test the hypothesis whether the conformation of extra-cellular domain becomes an active form by modifying the structure of intracellular domain mimicking platelet activation.

There are several clear limitations in our study. We have adopted the previously published crystal structure of the inactive form of integrin α _IIb_ β _3_ 47 as the extracellular domain of our integrated model of integrin α _IIb_ β _3_ . However, the crystal structure may not be identical to the functional structure of integrin α _IIb_ β _3_ in the human body. Moreover, the structure of transmembrane and intracellular domain was adopted from the prediction from NMR, electron cryo-microscopy, and single particle image reconstruction. 29 30 The precise structure of the transmembrane domain, especially α _IIb_ integrin was hard to be determined in a biochemical manner. 48 MD simulation revealed positional fluctuations of amino acids in integrin α _IIb_ β _3_ as shown in the attached Supplemental movie 1 and movie 2 (available in the online version). The results shown in the figures in this paper only reflect the snapshot of the fluctuating structure. Accordingly, we are not aiming to provide a new structural model as compared to the previously established ones. 49 50 Our goal in this paper is to show the persisting structural fluctuation of integrin α _IIb_ β _3_ even after convergence of MD calculation starting from inactive extracellular conformation. The use of the lipid membrane composed only from POPC without POPS may also influence the experimental results. Biological experiments revealed that the position of POPS changed from the inside to the outside of the platelet membrane. 51 However, the precise location of POPS in the membrane is still to be elucidated. While the initial structure did not contradict with previously published findings, 48 52 53 one may argue that our model was artificially developed even though we followed the previous publication to construct the integrated model. 29 30 To quantify the structural fluctuations of atoms constructing α _IIb_ β _3_ , the RMSDs were calculated in our study. However, the RMSD values may be influenced by errors such as inappropriate selection of initial structures. 54 The highest value of RMSD shown in extracellular domain of α _IIb_ may reflect the largest structural difference between the initial and calculated structure in the extracellular domain of α _IIb._ Despite these limitations, our major findings showing fluctuations even after the convergence of the integrated model is not influenced.

In conclusion, an integrated model of intracellular, transmembrane, and extracellular domain of integrin α _IIb_ β _3_ was developed on a computer. Molecular dynamic simulation calculation on our model suggests persisting structural fluctuation of integrin α _IIb_ β _3_ with inactive extracellular conformation incorporated into lipid membrane even after the convergence of MDs calculations.

## Notes

**What is Known on this Topic?**

- The structure and functions of the extracellular domain of platelet integrin α _IIb_ β _3_ (platelet membrane glycoprotein: GPIIb-IIIa) change substantially upon platelet activation.
- The talins and kindlins binding to the intracellular domain of integrin β3 are necessary to transform the extracellular domain of integrin αIIbβ3 into active forms.
- Molecular dynamic simulation can provide a clue to understand the biological functions of integrin *α _IIb_ β _3._*

**What Does this Paper Add?**

- The integrated model of platelet integrin *α _IIb_ β _3_* (platelet membrane glycoprotein: GPIIb-IIIa) constructed from intracellular, transmembrane, and extracellular domain suggested structural instability of integrin α _IIb_ β _3_ even in the inactive conformation of extra-cellular domain.
- Our calculation results are in agreement with previous publication but still provide hypothesis to be tested in the future.
